# Payday, ponchos, and promotions: a qualitative analysis of perspectives from non-governmental organization programme managers on community health worker motivation and incentives

**DOI:** 10.1186/1478-4491-12-66

**Published:** 2014-12-05

**Authors:** Marie-Renée B-Lajoie, Jennifer Hulme, Kirsten Johnson

**Affiliations:** Department of Family Medicine, McGill University, Montréal, QC Canada; Emergency Department, Jewish General Hospital, Montréal, QC Canada; Department of Community and Family Medicine, University of Toronto, Toronto, ON Canada

**Keywords:** Community health workers, Community health volunteers, Incentives, Programming, Health policy

## Abstract

**Background:**

Community health workers (CHWs) have been central to broadening the access and coverage of preventative and curative health services worldwide. Much has been debated about how to best remunerate and incentivize this workforce, varying from volunteers to full time workers. Policy bodies, including the WHO and USAID, now advocate for regular stipends.

**Methods:**

This qualitative study examines the perspective of health programme managers from 16 international non-governmental organizations (NGOs) who directly oversee programmes in resource-limited settings. It aimed to explore institutional guidelines and approaches to designing CHW incentives, and inquire about how NGO managers are adapting their approaches to working with CHWs in this shifting political and funding climate. Second, it meant to understand the position of stakeholders who design and manage non-governmental organization-run CHW programmes on what they consider priorities to boost CHW motivation. Individuals were recruited using typical case sampling through chain referral at the semi-annual CORE Group meeting in the spring of 2012. Semi-structured interviews were guided by a peer reviewed tool. Two reviewers analyzed the transcripts for thematic saturation.

**Results:**

Six key factors influenced programme manager decision-making: National-level government policy, donor practice, implicit organizational approaches, programmatic, cultural, and community contexts, experiences and values of managers, and the nature of the work asked of CHWs. Programme managers strongly relied on national government to provide clear guidance on CHW incentives schemes. Perspectives on remuneration varied greatly, from fears that it is unsustainable, to the view that it is a basic human right, and a mechanism to achieve greater gender equity. Programme managers were interested in exploring career paths and innovative financing schemes for CHWs, such as endowment funds or material sales, to heighten local ownership and sustainability of programmes. Participants also supported the creation of both national-level and global interfaces for sharing practical experience and best practices with other CHW programmes.

**Conclusion:**

Prescriptive recommendations for monetary remuneration, aside from those coming from national governments, will likely continue to meet resistance by NGOs, as contexts are nuanced. There is growing consensus that incentives should reflect the nature of the work asked of CHWs, and the potential for motivation through sustainable financial schemes other than regular salaries. Programme managers advocate for greater transparency and information sharing among organizations.

**Electronic supplementary material:**

The online version of this article (doi:10.1186/1478-4491-12-66) contains supplementary material, which is available to authorized users.

## Introduction

Community health workers (CHWs) have been central to broadening access and coverage of preventative and curative health services worldwide for over 50 years [1, 2]. More recently, human resource limitations in the Millennium Development Goals era have spurred a renewed focus on ‘task-shifting’ primary care responsibilities to lower-level health workers [3]. From volunteers to employees, their role varies between clinical providers and mediators of the health care system to agents of community empowerment. They are accountable to both the organizations and the communities they work in, with vastly different sets of obligations, expectations and recognition. This tension has been central to the longstanding debate about how best to incentivize and remunerate CHWs in order to motivate and support their work [4].

After decades of diverting CHW cadres as agents of vertical programmes, there is renewed interest in the core principle of Alma Ata of strengthening health systems as a whole [5–7]. The President’s Emergency Plan for AIDS Relief (PEPFAR) and United States Agency for International Development (USAID) have committed ‘to increasing the number of functional CHWs serving in maternal, newborn and child health (MNCH) priority countries by at least 100,000 by 2013’ [8]. There is growing interest in sharing best practices to develop, monitor and evaluate sustainable community-based programmes. This has revealed discordant views on how to define the role of CHWs within health care systems, and appropriate incentives for their services. Until recently, few organizations - or even Ministries of Health - have chosen to be prescriptive given the wide range of successful programme models.

This tension is in part due to the nature of the work of CHWs, often first a mediator between communities and the health care system, but also because of the complex nature of the relationship between incentives, motivation and performance. Motivation is defined as ‘the act or process of giving someone a reason for doing something’ [9]. In a human resource management context, incentives are the rewards - financial, material, and non-material rewards, including recognition - that motivate a desired behaviour or performance [10]. Motivational theory was recently popularized by social scientist Dan Pink. After reviewing the large body of research on the complex interplay between intrinsic (internal motivators such as altruism) and extrinsic incentives (external motivators such as money), he defines ‘autonomy, mastery and purpose’ as the pillars of motivation, and proposes that payment or performance-based incentives have the potential to extinguish intrinsic motivation, diminish performance, and crush creativity [10]. This may have practical applications in the CHW context [11]. Some studies of CHW cadres suggest that while financial incentives do not necessarily crowd-out intrinsic motivation, non-financial incentives are more effective at bolstering motivation [12]. Others demonstrate that pro-social, altruistic, personal growth and religious motivations can coexist with hopes for employment, or career aspirations for the future [13–19].

The most thorough review of CHW incentives to date, by Bhattacharya *et al.*, identified a number of key non-monetary elements that foster motivation, including community embeddedness, formal recognition, frequent supervision, training opportunities, a supportive political environment, and connecting the person’s work to a higher purpose [12]. Intrinsic incentives promoted a sense of self-worth and value, in tandem with extrinsic incentives, both monetary and non-monetary [12]. Material incentives included salaries, *per diems*, drug sales, and pay per performance. They found evidence that attrition rates were generally higher in volunteer programmes [20], but those paid inadequate or inconsistent stipends also experienced high attrition [20–22]. Non-material incentives, including preferential access to credit, health care, and inclusion in development programmes, were highly effective motivators. Job aids, including T-shirts, backpacks, bicycles and agricultural tools were found to promote pride, group solidarity and facilitate entry into households. Government ministries and non-governmental organizations (NGOs) alike struggled with how to sustain financial incentives. Payment also required exceptional supervision systems and accountability, but this sometimes worked to strengthen programming as a whole. The review identified a number of major gaps in operational research, including the cause of retention and dropout rates; effect of innovative financing and credit schemes; and the value of career paths for CHWs.

There are a few, often cited examples of where CHWs have been fully integrated into national health care systems and paid a salary, including the Brazilian Family Health Programme, the Ethiopian Health Extension Programme, and the Malawian Essential Health Package [23]. The Kenyan experience highlights the challenges in mandating financial remuneration for CHWs: the National Health and Human Resources plan initially recommended a standard minimum wage for CHWs, but later entered a clause that stated that this was dependent on the resources available to partner organizations, recognizing that the national government did not have sufficient funding [24]. High-resource settings also struggle to define CHWs as ‘employees’ versus agents ‘of the community’ [25–28]. In Massachusetts for example, CHWs recently established board certification to obtain recognition as health care workers and allowing them to advance in the workforce, a move which was highly controversial [29].

Since the Bhattacharya *et al*. review was published in 2001 [12], a number of key policy documents have opted to more narrowly define appropriate CHW incentives. The WHO, which previously had left remuneration to the organization’s discretion [30], now suggests that while volunteers make valuable contributions to short-term or part-time labour, formal remuneration is necessary for the long-term sustainability of CHW programmes [23]. They propose that adequate wages help retain human resources for health, especially in rural areas and among marginal communities, and may contribute to broader human development and poverty reduction strategies. The 2008 recommendations explicitly state that ‘*the burden of evidence indicates that stipends, travel allowances and other non-financial incentives are not enough to ensure the livelihood of health workers and that the absence of adequate wages will threaten the effectiveness and long-term sustainability of community health worker programmes*’ [31] *.* The NGO Code of Conduct for Health Systems Strengthening, a formal statement signed by leading international and national NGOs, called for NGOs to ‘*advocate for fair monetary compensation for work done by all employees, across the health care system, including salaries for community health workers*’ [32]. The USAID toolkit to assess the functionality of CHW programmes employs similar language to suggest that incentives be ‘*balanced, with both financial and non-financial incentives provided, and are in line with expectations placed on CHWs; for example, number and duration of visits to clients, workload, and services provided. Incentives are partially based on performance relevant to expectations, and include advancement opportunities and/or certification; community offers gifts or rewards*’ [33]. While the language remains purposefully vague, it is the first recommendation of its kind to add the consideration of workload and the type of services provided, as well as the importance of career opportunities for CHWs.

The extent to which governments and NGOs have responded to these policy recommendations is unclear. Compelling arguments for better compensation for CHW labour continue to surface in the qualitative literature [13–19]. This arena is often polarized between those who see CHW payment for labour as both an ethical imperative and a gendered social justice issue - where men are paid salaries and females are asked to volunteer - and those whose focus remains on non-monetary incentives and worry that wages undermine the altruistic voluntary nature of ‘lay health advising’. There is also concern that the WHO [31] recommendations risk overemphasizing payment, and skew the focus away from effective material and non-material incentives [34].

This qualitative study examines the perspective of international NGO health programme managers who directly oversee CHW programmes in resource-limited settings. It aims to draw on their common experiences in designing and implementing CHW incentives schemes in order to generate and sustain motivation. This study has two objectives. First, to explore institutional guidelines and approaches to designing CHW incentives, and inquire about how NGO managers are adapting their approaches to working with CHWs in this shifting political and funding climate. Second, to understand the position of stakeholders who design and manage NGO-run CHW programmes on what they consider priorities to boost CHW motivation.

This is the first study to specifically inquire about how NGO managers perceive the concepts of motivation and sustainability in the current political and funding context, and to explore the role of institutional, international and national policies in influencing programme managers and their programme design. Understanding current practices and existing tensions will help inform CHW programme design and policy recommendations at both the local and international level.

## Methods

Qualitative inquiry [35] was chosen to generate detailed descriptions from NGO programme managers about their experience, perceptions, tensions, intentions and behaviours surrounding CHW motivation at both the local and international policy level. The study had ethics approval from the McGill University Faculty of Medicine. Verbal informed consent was obtained from each participant. Pseudonyms were used during transcription to ensure anonymity.

Study participants: individuals were recruited using typical case sampling through chain referral at the semi-annual CORE Group meeting in the spring of 2012 [36]. All participants worked for affiliate member NGOs of the US-based CORE Group, a network of NGO and government partners that shares best practices and programmatic expertise in community-focused public health practice to impact maternal and child survival.

Data collection: a peer-reviewed semi-structured interview guide was developed based on an extensive search of peer reviewed and grey literature on CHW motivation and incentives. The central themes identified in the Bhattacharyya *et al*. [12] review formed the framework for the guide. The tool was then circulated among well-known topic experts with international NGOs and USAID for their feedback (Additional file 1). In total, 17 in-depth, semi-structured interviews were conducted until saturation on prominent themes was achieved. Consent was obtained to tape-record the sessions and the interviews were transcribed and, where they were conducted in French, translated into English by JH.

Data analysis: two of the authors (JH and MRBL) analyzed and coded the transcripts through multiple readings to identify meaningful patterns, first independently, and then together, using TAMSanalyzer software to organize the data [37]. Thematic content analysis was an iterative process of identifying major themes and identifying points of discordance between the two researchers for consideration.

## Results

A total of 17 programme managers were interviewed from 16 NGOs. Both expatriates and locally-hired programme managers were interviewed, with programme management positions ranging from one programme in one country, to multiple programmes across different nations; a few were based in their organization’s regional or central headquarters. All had experience in CHW programmes prior to working with their current organizations, and all had experience with directly managing country-level community health worker programmes.

Informants identified several key, often-dissonant factors that influence how incentives are developed (Additional file 2): organizational structure (available resources and implicit policies), context (programmatic, cultural, community dynamics), experiences and values of programme managers, the nature of the tasks asked of CHWs, and both donor practices and national-level government policy. These influencing factors are embedded here under the umbrellas themes of: national policy and health systems context; monetary; and non-monetary incentives for ease of discussion around these topics.

### National policy and heath systems context

All informants looked to national policies and norms to first guide their design of CHW incentive packages. They described this as both their personal and institutional approach to determining CHW incentives, with a few exceptions of organizations that pushed the envelope in terms of what countries will allow (them) to pay CHW’ (Informant 4). Many called for standardized national systems; they voiced that Ministries of Health and Finance should have a more prominent leadership and regulatory role to help standardize how NGOs work with CHWs in the country, and increasingly saw their role as collaborators with national and district governments. A few advocated for a ‘systems approach’ , consisting of multiple cadres of health workers, chosen by the community, with equity between equivalent-level workers, and a in-built career ladder. They felt this would avoid vertical programmes competing for CHWs with vastly different incentive schemes.

At the same time, informants offered examples where central governments have attempted to standardize CHW programmes by setting minimum salaries - either provided by the central government or set as a minimum for NGOs to pay CHWs - with minimal impact or unintended consequences. One programme manager voiced concern that CHWs were underperforming, despite nationally-set salaries, which threatened the long-term sustainability of the programme and ‘*undermined the ownership of the* (*…*) *messages*’ (Informant 11). Another CHW programme was described as a ‘*really low paid civil servants programme … they do just the minimal amount and get their money*’. In these cases, many reported that NGOs ‘top up’ these salaries, that is provide extra incentives in addition of the recommended wages, in an attempt to increase motivation and performance, especially within HIV programmes (Informant 4). One West African country had recently announced that all NGOs had to pay a set salary to all CHWs, with major concerns raised by programme managers: ‘*e would have given bicycles, supplies, been able to increase supervision and support, plenty of job aids, T-shirts, badges … Would’ve given everything! But now the budget is so restricted that we’re unable to provide a lot of these things and the money will go into salaries*’ (Informant 5).

Underlying the discourse on the role of national leadership and guidelines was the mandate of the NGO itself in building the capacity of the national health systems: ‘(*Our*) *objective is not to provide jobs for community health workers; our goal is to reduce maternal and child mortality*’ (Informant 14). Programme managers described inherent tension between their programme objectives, donor-driven decisions, and the theoretical goal of supporting the Ministry of Health objectives and health system strengthening.

Inequity, inconsistency, and competition between programmes in the context of short-term funding cycles were cited by most as major barriers to sustainability, long-term motivation and effective human resource capacity building in CHW programmes. The influx of short-term programmes within unequal incentives were described as ‘*destructive*’ and ‘*damaging’* , and threatening the integrity of long-term CHW programmes - both government and NGO-supported - and subsequently, health system strengthening as a whole.

Informants asked Ministries of Health and donors to support long-term strategic objectives in CHW programmes to avoid the current tension between short-term programmatic goals and the long-term viability of CHW programmes. Programme managers requested a centralized information data source, organized by country or regions, to document experience to date with CHW programmes. This platform would showcase high-quality operations research, and work to share local experience with monetary and non-monetary incentives, and document existing programmes.

### Monetary incentives: varying perspectives

#### Pay for work as unsustainable

Informants spoke from their experience seeing CHW-rooted programmes collapse after financial incentives were withdrawn: ‘*If the Ministry of health couldn’t support a system like that, why would you ever start one? We can’t do what we’re doing without* (*CHWs*)’ (Informant 14)*.* Short-term or one-off payments from other international agencies also were viewed as undermining long-term objectives of CHW programmes. Many described resistance from donor agencies to incorporate financial incentives if they could not be sustained after the close of the programme, highlighting the implicit impact of donor practices on incentive design. The true cost of paying large cadres of CHWs regular stipends were an ‘*enormous amount*’ (Informant 17), and ‘*expensive*’ (Informant 15)*.* This pushed programmes to ‘*move away from payment or incentives to other forms of compensation that may be more sustainable*’ (Informant 15)*.*

There were a number of problems that were specific to salaries as the chosen from of financial remuneration. ‘*Salaries are never enough*’ (Informant 11), and yield collective power to cadres of CHWs who may try to renegotiate their pay, and in one instance, went on strike. Others countered that ‘*it is human nature*’ and CHWs will always ask for more incentives, describing instances where transport costs or bicycle repairs had to be renegotiated. Programme managers were alerted to their dissatisfaction indirectly through the CHWs’ direct supervisors, or when they themselves made field visits. Several informants explicitly distrusted the motivations of salaried CHWs: ‘*They may actually ensure that there are some resistant families so that they continue to have a job!*’ (Informant 8); ‘*people need to extract as much out of* (*the programme*) *as possible*’ (Informant 17)*.* One participant felt that money attracted men more than women, with potentially the ‘wrong’ motivations:

‘*If you make something a well-paid job it tends to attract men, and you push women out of the equation. Because if they could be in a full-time job, they already would be. So now you’re attracting men to the job who are motivated by the salary, but are not necessarily motivated by the right things. And there you get your difference in performance*’*.* (Informant 15)

All informants unambiguously stated that pay is insufficient to motivate performance, and highlighted that ‘*… there are people who keep your money and they move on. There are community health workers who are really doing nothing. So motivation is complex*’ (Informant 5).

### Pay for work as a right

A number of informants were critical of the discourse around CHW stipends being unsustainable, citing the ongoing fixation on volunteerism as ideological, ‘*almost a religion*’ (Informant 4). Their language was impassioned and they equated monetary compensation with respect (Informants 3, 4, 8, 12 and 16):

‘*It’s not spoiling someone to give them a salary. You are economically empowering them and they will probably do a better job. Why should we have different standards for them and us. If I’m fired from my present job than I will look for something else.* (*…*) *If they were men - why are there no male community health volunteers? So why are women expected to work for free?*’ (Informant 8)

In addition to gender bias, many illustrated that there was also a double standard implied with paying some workers and not others: ‘*I hate the idea that this is about someone’s heart’s calling. That means that you and I should also not be paid if this is our heart’s calling*’ (Informant 3)*.*

While many other informants rejected blanket recommendations of salaries, many expressed guilt from having managed programmes where CHWs were overworked, undercompensated, or both, and wished they could have done more to recognize and compensate them at that time: ‘*I met a community health worker the other day and he explained how many hours a day he spent on his role, which meant that he was doing two jobs, and then it felt more difficult that we weren’t giving any money for his job. His time was precious, he has nine children, I felt quite badly actually that we weren’t giving anything for that part of his time*’ (Informant 13)*.*

### Middle ground: incorporating context and the burden of work

Other informants did not have set ideas about remuneration for CHWs, and described an evolution in their thinking over the course of their careers. Several described starting their careers believing that all CHWs should be paid salaries, and now felt that there is no one size fits all; that incentives should be adapted to historical, cultural, and contextual realities: ‘*At a time when they were huge number of people infected with treatable infectious diseases* (*…*) *these questions we had to cut with a blunt knife. We said “we need to pay them, we need to get value, we need to get these programmes scaled up”. Now I think* (*…*) *there are subtle areas. I do think that there are different cadres that can be incentivized with different mechanisms*’ (Informant 4). Even those informants whose organizations or personal philosophies championed monetary incentives as a right didn’t necessarily view money as the root of motivation and high performance: ‘(*Money is*) *never enough.* (*…*) *But does this really motivate community health workers, that is still the question*’ (Informant 9).

Some of the considerations included the nature of the work itself. Respondents described the nature of some CHW work where they have set targets, little control over their time, or ‘*something that interferes with people’s daily lives*’ (Informant 15) as the type of work that requires monetary compensation (Informants 2, 4, 13, 10 and 15). ‘*If you have somebody working every day, going house to house, giving the medicine, going back and forth to the clinic … then the salary makes more sense*’ (Informant 4).

Nearly all participants voiced the importance of local and national context in making recommendations for CHW incentive packages across and between countries and regions. There were concerns that CHWs were overworked and undervalued in the context of constantly changing incentives and programmatic objectives.

One informant reiterated the fact that the long history of one programme is what makes their success possible today: ‘*Now we don’t appreciate what it was like before the road was built. So right now if you have a small pothole, you complain a lot. We don’t imagine what it was like when there was no tarmac. What the vitamin A programme did - is it paved the road*’ (Informant 2). Given the nuance of the history of each CHW cadre and difficulties in defining competencies and workload, there was pervasive discomfort with the idea of universal guiding principles and blanket descriptions to the development of effective programmes as ‘*there are lot of variables in terms of satisfaction’* (Informant 4), ‘*different situations, different programmes, and different contexts*’ (Informant 2). ‘ *… what a community health worker is doing in Afghanistan, Mongolia, or urban versus rural sub-Saharan Africa is really quite different*’ (Informant 1).

### Alternative financing schemes

Many informants expressed interest and positive experiences with less traditional forms of monetary support for CHWs, especially in the cases where a CHW may not have extensive daily tasks or data collection duties that might require salaried work. Informants cited community-based microfinance programmes, small enterprise training, and drug and other material sales as more sustainable incentives that, in some cases, may prove to be more motivating, particularly if it ‘*allows them to earn money down the line* (*while) contribut*(*ing*) *to their community*’ (Informant 17); ‘ *… where people got drugs that they can sell, they get recognition from their communities to be the special envoy to any special guests to the area and special recognition*’ (Informant 16).

An informant familiar with the Female Community Health Volunteer (FCHV) programme in Nepal elaborated on a series of income-generating and financing schemes that linked community health volunteers to existing income-generating projects, and developed a savings credits fund, financed by community leaders, which later evolved into a emergency loans fund for FCHVs that could be used for their personal and small business emergencies. Expansion of their responsibilities to include community-based treatment of pneumonia was seen to warrant a monetary incentive, ideally through a self-renewable source of financing. See Additional file 3 for the story in his words.

### ‘It takes more than money’: non-material incentives

Programme managers agreed that status, recognition, and a genuine sense of purpose in their jobs were the most important tenants of CHW motivation, and by extension, of their performance, satisfaction and retention. Managers explicitly helped CHWs see the bigger picture early on in the project, and involved them in the design and roll out of the programme so that they ‘*buy into the objectives of the programme*’ (Informant 16). They found that explicit efforts to engage CHWs as growing professionals, including activities such as ‘*training their peers*’ and ‘*celebrating* (*their*) *knowledge*’ (Informant 15), resulted in a more active, engaged cadre of health workers.

Recognition took many forms. Material incentives like badges, T-shirts, certificates and job aids helped CHWs directly to help gain recognition from their own communities. Informants referred to broader strategies to recognize their work, through regular supportive supervision, the use of radio announcements, ceremonies, or representation on committees together with local government. Public recognition alongside celebrities or respected politicians was a deliberate strategy to heighten the credibility of female CHWs, and CHW programmes were seen as an opportunity to empower women. While little was mentioned in regards to community recognition, informants described their role as managers in affording recognition: ‘*Just being as humble and grateful as possible, and simply recognize how difficult it this work is, and how far even that can go. But it has to be consistent, and it has to be genuine*’ (Informant 10)*.*

Inconsistent management and oversight was a major challenge in programmes. Insufficient and irregular supervision negatively affects motivation, and programme managers found it difficult to measure performance and keep mid-level managers and CHWs accountable in their programmes. Several informants suggested that regular stipends could work to improve supervision, with the advantage of being able to dismiss under-performing CHWs if needed. However, this doesn’t negate the essential role of supervision and support, and ‘ … (*they*)*’re not always doing the follow-up that* (*they*) *should.* (*They*) *should be doing visits with them to see how they’re doing it. It’s time-intensive*’ (Informant 7).

## Discussion

A wide range of CHW competencies, roles and responsibilities were reported, making it challenging to recommend universal best practices in CHW motivation and incentive schemes. Out of key factors that influence CHW incentives - including national-level government policy, donor practice, implicit organizational approaches and structure, local context, experiences and values of managers, and the nature of the work asked of CHWs - donor practices and what other organizations and governments were offering CHWs had the biggest influence on NGO practices, particularly in defining what is ‘sustainable’ beyond the duration of the programme. Surprisingly, with a few notable exceptions, organizational policy and philosophy did not consistently drive most organizational approaches to CHW programming, and programmes were often developed on a case-by-base basis. Other key considerations in developing incentive packages were the desired programmatic outcome, the workload and opportunity cost for the CHW, and the nature of the work itself. Once these elements are addressed, NGOs described a mix of extrinsic incentives - including both monetary and non-monetary material incentives - and intrinsic incentives targeted at heightening CHW recognition and their sense of purpose.

Many of these findings underpin the best practices in CHW programming that have been described elsewhere [12]. This study also has important limitations, as we did not consult communities and affected CHWs directly. However, this study highlights the perspective of the NGO community, with a number of health policy issues that merit careful consideration by governments, donors and NGOs alike. The destructive nature of one-off payments and inequality of incentives between programmes profoundly affects NGO-supported and government community health worker cadres. The lack of central oversight and pragmatic forums for information sharing between organizations, and the persistent myopia of short-term donor targets, were all cited as culprits. Programme managers are seeking guidance from the Ministries of Health in their initial approach to supporting CHW programmes and dynamic online forums for information sharing on CHW motivation between organizations and governments. This presents an opportunity for governments to recognize CHWs as a major force in their strategies and their central role in addressing health system strengthening through partnerships with NGOs. This would also strengthen the central tenants of supervision, training, recognition, and possibly career paths for these cadres of workers.

Payment has resided at the heart of recent debates in the social science literature, WHO recommendations, and the NGO health systems code of ethics [6, 13, 23, 30, 31, 38, 39]. Guilt, gender, the ethics of payment for work, and increasingly overburdened CHWs all emerged as important themes in this work. Many informants at one time felt that their programmes provided inadequate remuneration or support for CHWs on the whole. The problems with cash stipends raised here are also not new. Inconsistent remuneration and fluctuating in-kind payments both work to demotivate volunteers [6, 22]. The inequitable and variable distribution of incentives between regions and programme was also cited as an important problem in this study, and this has been documented elsewhere [40]. Perhaps the most interesting paradox described in this study is that the potential of financial compensation to empower community workers, especially women, coexists with managers’ distrust of their motivations and fear that payment will give CHWs the power to arbitrate for more compensation.

Incentives should remain context dependent, according to many informants, with the *caveat* that different types of community-based health work may be best suited for different types of incentives. This is consistent with the recent discourse surrounding CHW compensation, which underlines that a baseline wage is needed when a volunteer cannot be expected to do the job [11, 37]. What is ‘too much work’ for a volunteer relies not only on the autonomy and flexibility in job hours and responsibilities, described above, but overall keeping the workload light. Informants almost universally cited door-to-door visits, Directly Observed Therapy Systems (DOTS) with targets, and data collection as examples of job descriptions that minimize the control CHWs have over their time, and merit monetary compensation. Our findings are in line with recent recommendations put forth by Tom Davis, a US-based thought-leader in this area, who put forth eight hours a week as a maximum number of hours that a volunteer should work, and advocates that if you are going to pay CHWs, ‘give them a lot to do, and pay them a fair wage’. He also submits that a middle ground, with some small non-financial incentives and low pay, might indeed be the most dangerous motivational set-up, where CHWs are resentful of an insulting, inadequate wage [11, 22].

This leads to the expanding vision for fair monetary and non-monetary incentive models, which considers innovative approaches to monetary compensation, and much more purposeful investment in human resources, including continuing education, skills-training, and career ladders that honour the individual’s desire to grow in their role, and move into other forms of employment. Perhaps the most illuminating example was from the Female Community Health Volunteer (FCHV) programme in Nepal, which has been hotly debated in the social science literature, polarized between the perspectives of male salaried public servants on the cultural merits of volunteerism, versus those concerned about the gender bias inherent in female volunteerism and the potential for exploitation [34, 17]. On closer inspection, we learn that regular demands on FCHVs are typically less than five hours a week, and more demanding tasks, such as an immunization day, *are* financially compensated. FCHVs also benefit from a ‘credits and savings programme’ , which informants considered a key factor in helping sustain their motivation. These elements are notably absent in the debates around ‘volunteerism’. Irrespective, these discussions serve to highlight the complexity of investing in this essential tier of human resources for health.

## Conclusion

This qualitative study provides unique insight from NGO programme managers on the debate surrounding how best to support CHW motivation. It highlights the central role of Ministries of Health to drive national standards and appropriate motivation schemes; NGO partners are increasingly committed to supporting national programmes and policies, despite the short-term vision of their donor-driven outputs. Our findings suggest an expanding vision for fair monetary and non-monetary incentive models, which will necessitate thoughtful investment in human resources, including continuing education, skills-training, and career ladders that honour the individual’s desire to grow in their role, and potentially move into other forms of employment. Participants called for high quality operations research and documentation of current practices in order to continue to address challenging health needs, and strengthen existing community health systems.

## Electronic supplementary material

Additional file 1: **Semi-structured questionnaire outline.** (DOCX 16 KB)

Additional file 2: **Key influencers of CHW incentive strategies.** (DOCX 22 KB)

Additional file 3: **Key informant narrative: the evolution of incentive strategies for CHWs.** (DOCX 15 KB)

## Abbreviations

CHW: Community health workers
CORE: Community
HIV: Human Immunodeficiency Virus
DOTS: Directly Observed Therapy Systems
FCHV: Female Community Health Volunteer
NGO: Non-governmental organizations
MNCH: Maternal, newborn and child health
PEPFAR: President’s Emergency Plan for AIDS Relief
USAID: United States Agency for International Development
WHO: World Health Organization.
