# Supplementary material for: Payday, ponchos, and promotions: a qualitative analysis of perspectives from non-governmental organization programme managers on community health worker motivation and incentives
Source: Hum Resour Health. 2014 Dec 5;12:66. doi: 10.1186/1478-4491-12-66 (PMC4267436; doi:10.1186/1478-4491-12-66)
Supplement: Supplementary file 1 — Additional file 1: Semi-structured questionnaire outline. (DOCX 16 KB) [file 12960_2014_461_MOESM1_ESM.docx]

Additional file 1

Semi-structured questionnaire template

- Does your organization have any general guidelines (written or unwritten) on how they go about developing and choosing when to use different CHW incentive packages?
- Are you aware of any recent changes in how your organization approaches CHW incentives and motivation? How has your thinking about incentives changed over the past few years? What has prompted these changes in how you use incentives and how you think about them?
  - Have you received information that changed your thinking about incentives (e.g., books, videos, blogs, policy documents)? If so, from what source did you receive that information, and what information did you receive?
  - Have program budgets been significantly affected by these changes? How so?
  - Do they address career paths or opportunities consistent with professional advancement? What does this consist of?
- Do you think that there should be certain universal principles that apply to developing or choosing an incentive schema for CHW programs?
  - (If yes) What sorts of guidance or rules would you like to see emerge as universal principals?
  - What should they be based on? (Prompts) The number of hours worked? The type of activity performed? (e.g., counseling versus DOTS/ community case management)
- In general, who influences policy decisions regarding CHW incentives? (i.e. at the institutional and country level)
  - In your experience, ideally who should be responsible for setting incentive packages to avoid discrepancies and competition?
    - (Prompts) Individual organizations, the donor community, national governments, other regional or national policy agencies?

- In your position, what would be the most useful guidelines or information for you in designing incentive and motivation programs that support CHWs?
  - (Prompts) Do you need information on ***when*** to use each type of incentive? Scientific information on ***how*** different types of incentives work and which incentives have been found to work in different settings or for different tasks? **Information on how *other organizations*** are using as incentives and their experience with using incentives? Information on **sustainability** and incentives?
- Do you directly oversee a country program that supports Community Health Workers? (If yes)
  - What are the strengths and weaknesses of the CHW incentives in the programs with which you work, in your opinion?
  - Do you think that incentives provided in that program are reasonable and sufficient?
  - For what types of work or tasks do you think non-monetary/material incentives work the best, if any?
  - For what types of work or tasks do you think monetary / material incentives work the best, if any?
  - What types of monetary or material incentives have you found work the best?
  - What types of non-monetary / non-material incentives have you found work the best?
  - Do you help CHWs to think about their work or themselves in certain ways in order to help them stay motivated? If so, what do you do in that regard?
  - Do you provide opportunities for CHWs to gain professional advancement as incentives, such as continuing education or opportunities for promotion? How has this worked in practice?
- Have CHWs in any of your programs actively voiced concern or dissatisfaction with their job conditions, pay and/or incentives? To what extent? If CHWs are dissatisfied with their tasks, pay, or the incentives that they receive, to whom do they usually turn?
- Is there anything else you’d like to tell me about how incentives have been designed or chosen, and how they have worked or not worked in your organization’s programs?
