# Supplementary material for: Payday, ponchos, and promotions: a qualitative analysis of perspectives from non-governmental organization programme managers on community health worker motivation and incentives
Source: Hum Resour Health. 2014 Dec 5;12:66. doi: 10.1186/1478-4491-12-66 (PMC4267436; doi:10.1186/1478-4491-12-66)
Supplement: Supplementary file 2 — Additional file 2: Key influencers of CHW incentive strategies. (DOCX 22 KB) [file 12960_2014_461_MOESM2_ESM.docx]

**Additional file 2: Key influencers of CHW incentive strategies**

| **Organizational structure: resources and implicit policies**  “[CHW incentives are determined] at the country level, and probably our program in one country would have some guiding principles. We try to do some… sharing about what's working here, ‘we tried this here’, and try to get other managers to do similar things.” (Informant 7)  “[Our NGO] just constantly pushes the envelope to see how much they will allow us to pay community health workers... in general we have a philosophy about that community health workers should be paid. But we have to adopt that to what local partners suggest and demand.” (Informant 4)  “…at the government level, international organizations like UNICEF the WHO, GAVI, these organizations are getting salaries to community health workers, and it's very destructive. They call them community extension workers, so for a vaccination campaign they pay them. When they go to the schools for a vaccination day for example, those community health workers that you're working with will ask for the day off, but we know that it's to work for the vaccination campaign.” (Informant 17) |
| --- |
| **Context: programmatic, cultural, and community dynamics**  “I'm not sure if there's a universal passage that we can recommend for all programs. It needs to be tailored to the country situation..” (Informant 14)  “Even local realities might not be the same. What's happening in [one country] might not be applicable to other settings. For example we have very mountainous communities, so a community health worker up in the mountains has to travel several kilometers to get from one community to another, as opposed to someone who works in the plains.” (Informant 3)  “…we learned that the hard way, because we wrote the proposal with someone who's experience was in Africa, where community workers are volunteers, and a T-shirt or a bicycle would be sufficient.. And then we got out into the communities, and they were like, ‘no, we've never done this for free’. So we had to cough up private resources to cover their stipends. Which was interesting.” (Informant 11)  “…and the men in Haiti have a huge catchment area, and then you see things fall into the cracks. Because they're simply not there for the follow-up and don't feel a connection to their population. You can have an unseen number of home visits, but if they don't feel that connection to their own population because they're catchment area so big then the impact won't be there. (Informant 15) |
| **Nature of CHW work**  “Certainly someone who is considered a volunteer who gets a badge or T-shirt showing that their volunteer to be able to go into a house is different than someone who is doing community-based treatment, and is considered a community health worker. Their incentive package would be very different but that's inherent in the program.” (Informant 14)  “I think there is a time and a place if you're expecting a lot of data collection and otherwise, for monetary incentives, depending on your donor and what you want to accomplish…. I think it's unjust and unfair to ask people to do tons of volunteer work that will negatively affect their own life and livelihoods. And they probably wouldn't do it anyway. But it would be wrong and unjust and unfair for us to expect that they would.” (Informant 10)  “Take the Dominican Republic and Haiti, where in the Dominican Republic they’re paid less and in Haiti there paid much more. This example really suggested to me that being paid less but working part time with more flexibility may actually be better than doing this kind of work is a full-time job per se. […] One off tasks can respond very well to nonmonetary incentives. Giving blood for example. You don't need to pay people to give blood because it doesn't interfere with their daily lives they do it once and they've filled a small duty and feel good about themselves. But when you're doing something that really interferes with people's daily lives, with a really helped with standard and are expected to perform, then you need some form of monetary incentive. Because that impedes their ability to make money in other ways and is interfering with their daily lives.” (Informant 15)  “…campaigns? Sure! Give T-shirts, that’s fine. But when you ask them to provide service that requires time, that required skills, knowledge, confidence support.… Giving ORS is different from giving cotrim, giving cotrim is different than giving …gentamicin,… But they think that because you are able to learn French you are able to learn any language.” (Informant 2)  “We need them to do a specific number of tasks during the day, especially when it requires a certain amount of complex thinking - then you have to think about monetary incentives. Like, for example, in sensitization for cholera treatment we need people to go to 10 households today. That's a lot of households. So how could we expect that to be on a voluntary basis? It's a job. They don't have control over their time.” (Informant 11) |
| **Experience and values of program managers**  “I initially started out thinking that absolutely everyone must be paid for the time. And I suppose now that I've been on the field for so long I realize that it's much more nuanced, and I don't think that everyone needs to be paid for community health work. There's no black and white, not always, I think it's purely contextual and you have to look at what's happening around you.” (Informant 10)  “But since I've been in this job, I guess initially I thought that all community health workers should be paid, should be paid well, and that this should be seen as a job. And seeing how works in the field is changed how I think about this.” (Informant 15)  “The problem above all else is that it’s not evidence-based. Everyone has his or her own opinion and it’s often ideological.” (Informant 4) |
| **Donor practices**  “In terms of the point of view of the donor, it is not reasonable that we resolve their community problems, and at the same time be paying them. We want to be paying them and we also want to be training them? This is not reasonable.” (Informant 17)  “The problem is of course that most of the things are driven by donor funding. There's a lot of money for HIV and there's no money for non-communicable diseases… if you have no funding at all, then you really do rely on volunteers. So it's all very well have the policies that say all community health workers are equal. But if the funding is channelled towards one particular disease condition than that's difficult to then marry up on the ground.”  (Informant 1)  “NGOs that I worked with in the past used to say that the donor doesn't want to give incentives to community health workers. Don't spoil them. We are funded a little bit by Bill Gates and USAID and have never had any issues with paying the mobilizers. So I don't really buy that…. It's really the way that you present it to the donors. “ (Informant 8)  “I think organizations should stop looking at this as if it's a big investment. They should start looking at the big picture […] For example, donors used to ask us to make projections based on what we were investing in CHWs per family planning user. It would look extremely expensive - it cost $100 for each family planning user. - but then 3 years later after the project was over we went back, and 50% of newcomers were still using a family planning method. They were getting information from their neighbours…. but there are no longer paid community health workers in that setting. So that uptake has taken place without direct investment. Eventually over the course of long long-term follow-up, what you paid to the community health worker is now diffusing in the form of knowledge.” (Informant 12) |
| **National level government policy**  “... of course our principles are to follow the national policy. The goal is transparency.” (Informant 9)  “The approach we've always had is to find out what is recommended by the national policy and follow the national policy...except that there's certain structural things that I can't change in terms of what the norm is on the ground... of course our principles are to follow the national policy. The goal is transparency. (Informant 9)  “[…] Right now we’re not even in a position to pay people yet because the guidelines from the government about how to pay them and what's the definition of high performance really has not been clear. And we've had to radically change our budgets to accommodate this new policy…. So it's very interesting… because they'll be a slow rollout where we can see if there's a real difference in motivation between those who have performance-based payments and those who do not. " (Informant 5)  ““I think you need to be very aligned with what the Ministry is doing… with South Sudan, it's part of the basic package of health services, but that's the idea: that everyone aims towards the same package […] We were so focused to getting it exactly like the Government policy should be, where as we don't necessarily have those pressures and other places. But we should, that would be best practice. We are not always making sure that it's done the way the government really would like it to be done. (Informant 7)  “The donors write a lot about what the government policies are, so ideally the donors are basing it on government policy, and it would've worked because everyone was used to the same system. But the NGOs violating this is like a slap in the face, even to the community health workers who really want to serve their community.” (Informant 16) |
