# Supplementary material for: Payday, ponchos, and promotions: a qualitative analysis of perspectives from non-governmental organization programme managers on community health worker motivation and incentives
Source: Hum Resour Health. 2014 Dec 5;12:66. doi: 10.1186/1478-4491-12-66 (PMC4267436; doi:10.1186/1478-4491-12-66)
Supplement: Supplementary file 3 — Additional file 3: Key informant narrative: the evolution of incentive strategies for CHWs. (DOCX 15 KB) [file 12960_2014_461_MOESM3_ESM.docx]

**Additional file 3**

**“Pneumonia is a very strong intervention…. you can motivate people very well to pay for that. And that’s when I started the whole concept of the endowment fund. […] So if you put Rs. 50,000 as a one-time investment they can do it! For 9 volunteers, Rs. 50,000. They said, ‘in a poor country, how can we bring the money?’ - and nobody put any money. Finally I convinced one of the local chiefs - he said “Oh I don’t have that kind of money”, so he put just Rs. 25,000. And then we got on the radio and TV saying, look! This is the village chief who gave this kind of money. Then, slowly, other village chiefs said ‘okay, no problem’. Just for 1 year, you can do something for your children…. so that’s how we convinced them. So right now all villages of Nepal have funding from the local government. The interest [from this central fund] will go to a savings account, and those 9 women will be responsible for that. I spoke to other organizations that were teaching savings and credit [courses], and asked, can you give skills to these women? …[The FCHVs then] said “we all know what to do with the money now. We want to have savings and credits”… So now any time you go to the village they will tell you about how they designed the saving and credits group… Everybody wants to see benefit. The village chief looks great because he can say ‘that’s what I did’. The women are benefiting because the money is coming to them. In every village, the volunteer has money. But it’s fixed capital. And nobody talks about it. And nobody writes about it.” (Informant 2)**
